# Choice and Duration of Anticoagulation for Venous Thromboembolism

**DOI:** 10.3390/jcm13010301

**Published:** 2024-01-04

**Authors:** Aroosa Malik, Nghi B. Ha, Geoffrey D. Barnes

**Affiliations:** 1 Department of Internal Medicine, Division of Cardiovascular Medicine, Frankel Cardiovascular Center, University of Michigan, Ann Arbor, MI 48109, USA; 2 Pharmacy Innovations & Partnerships, University of Michigan, Ann Arbor, MI 48108, USA; nghih@med.umich.edu

**Keywords:** venous thromboembolism, pulmonary embolism, deep vein thrombosis, anticoagulation, cardio-vascular disease

## Abstract

Venous thromboembolism (VTE) is a prevalent medical condition with high morbidity, mortality, and associated costs. Anticoagulation remains the main treatment for VTE, though the decision on when, how, and for how long to administer anticoagulants is increasingly complex. This review highlights the different phases of VTE management, with special circumstances for consideration such as antiphospholipid syndrome, coronary artery disease, cancer-associated thrombus, COVID-19, and future anticoagulation options. Anticoagulation management will continue to be a complex decision, applying evidence-based medicine to individual patients with the hope of maximizing effectiveness while minimizing risks.

## 1. Introduction

Venous thromboembolism (VTE) is defined as a blood clot in the venous system, occurring as a deep vein thrombosis (DVT) or pulmonary embolism (PE). The annual incidence of VTE in the United States is estimated to be around 1–2 per 1000 people, or 300,000–600,000 cases. However, the incidence is noted to differ by age, with VTE occurring in 1 per 100 people aged ≥ 80 years old. The estimated total annual healthcare cost for VTE ranges from USD 2–10 billion. The disease process carries high morbidity and mortality, with 10–30% of patients having a 30-day mortality. Additionally, 20–25% of PE cases present with sudden death [1]. Around 60,000–100,000 deaths occur annually from VTE. A third of the people who have a VTE event will have a reoccurrence within 10 years, while a third of the patients with DVT will develop post-thrombotic syndrome [1,2]. This highlights the high burden of VTE on the healthcare system and the importance of its management, including preventing recurrence.

Traditionally, most VTE events are characterized according to the presence or absence of provoking risk factors. Provoked events can be further characterized as a transient risk factor vs. persistent risk factor, while for unprovoked events, they have no provoking factor, either transient or persistent [3] (Table 1).

Venous thromboembolism management continues to be an evolving field with considerations in choice and durations for anticoagulation. This review will outline VTE management decisions, focusing on various anticoagulation options, treatment length, and special considerations.

## 2. Overview of Anticoagulation

**Figure 1:**
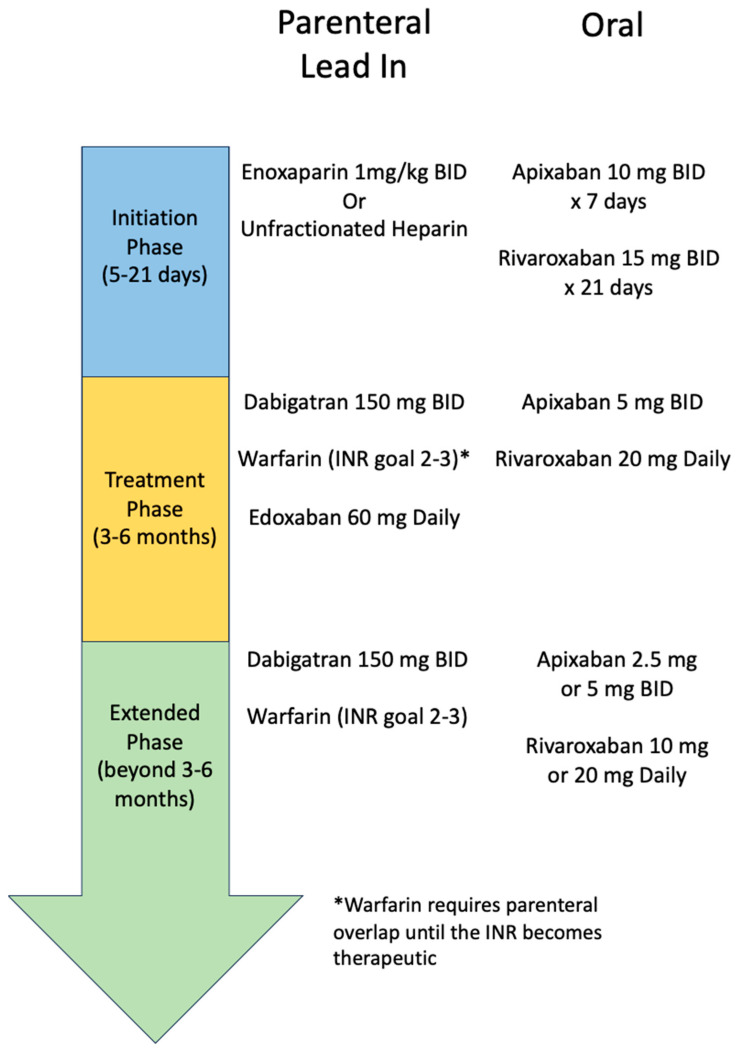
Choice and duration of anticoagulation for VTE.

Anticoagulation is the bedrock of VTE management, given its proven role in preventing VTE occurrence and recurrence. For nearly all patients with a proximal DVT or acute PE, anticoagulation is recommended as first-line therapy. The treatment for VTE is typically divided into three phases: the initiation phase, the treatment phase (primary treatment), and the extended phase (secondary prevention) (Figure 1). The goal of the initiation phase is to slow down any active thrombus formation, helping to prevent new thrombus from forming while allowing the body’s natural thrombolytic process to proceed and restore/maintain venous blood flow. This can be achieved through either oral anticoagulation, with apixaban or rivaroxaban, or through parental medication (e.g., unfractionated heparin, low-molecular-weight heparin). For the treatment phase, all patients are recommended to receive 3–6 months of treatment with anticoagulation. This is the time when patients are at the highest risk of recurrence as an acute thrombus is being converted to fibrin [4]. Given their overall improved safety profile (especially lower rates of intracranial hemorrhage) and ease of administration, anticoagulation with apixaban, dabigatran, edoxaban, or rivaroxaban is recommended over vitamin K antagonist (VKA) for the treatment phase [5] (Table 2). For patients with a continued risk of VTE they will continue anticoagulation in the extended phase. In this phase, the risk vs. benefit of full-dose anticoagulation vs. reduced-dose anticoagulation vs. no anticoagulation will need to be considered depending on the patient’s risk factors (Figure 1).

Apart from anticoagulation route selection, it is also important to decide where management should take place. For patients with a DVT, if there is rapid availability of ultrasound and ease of communication, then outpatient management is usually preferred. Exceptions would be for patients with a high risk of limb loss (e.g., phlegmasia cerula dolens) or an inability to reliably obtain anticoagulant medications in a timely manner and clinic follow-up. Most PEs are first evaluated in the emergency department where they are risk stratified for the risk of deterioration. Patients at low risk for complications should be offered an outpatient management strategy if there is appropriate availability of testing, medications, and clinical follow-up. A study has estimated that 20% or more of acute PE cases in the emergency department may be good candidates for outpatient treatment [6]. The remaining cases typically require a hospital stay.

An exception to routine anticoagulation for VTE treatment is in patients with distal DVT. Distal DVTs affect the deep veins, with the most proximal component being distal to the popliteal vein. The ninth edition of the CHEST guideline recommends serial ultrasound in 1–2 weeks without anticoagulation if the thrombus does not extend proximally. However, if there is extension into proximal veins, anticoagulation is strongly recommended [5]. Additionally, cases with distal DVT anticoagulation may be appropriate for patients with significant symptoms or a high risk of extension (e.g., underlying malignancy).

Another exception for routine anticoagulation treatment is in patients with isolated subsegmental PE without proximal DVT. In these patients, the risk of recurrence needs to be considered. In patients with a low risk of recurrent VTE, clinical surveillance can be considered, while for patients with a high risk of recurrent VTE, anticoagulation is recommended. These are both classified as weak recommendations by the most recent CHEST guidelines [5].

The third important exception is in patients with active bleeding, for whom anticoagulation should be avoided. It is also reasonable to consider avoiding anticoagulation for patients at very high risk of bleeding. The patient’s risk vs. benefit of anticoagulation needs to be considered in the setting of VTE management and bleeding, with continued re-assessment. If anticoagulation is not being pursued, then the role of an IVC filter should be reviewed.

## 3. Phases of Management of VTE

### 3.1. Initiation Phase

During the initiation phase, the goal is to stop the growth of the thrombus and prevent embolism of the thrombus with anticoagulation. This can occur over 5 to 21 days, depending on the anticoagulation chosen. Traditionally, unfractionated heparin, or low-molecular-weight heparin, was the anticoagulant of choice. Now, apixaban and rivaroxaban are oral options that can be used for the initiation phase. Typically, if patients are hospitalized in the acute setting for VTE, they are initially started on a parenteral heparin agent and then transitioned to oral options prior to hospital discharge. For parenteral heparin agents, low-molecular-weight heparin (e.g., enoxaparin) is preferred over unfractionated heparin given the lower risk of HIT, subcutaneous administration, ease of dosing, and most importantly, a predictable anticoagulation level without requiring routine monitoring [4]. When this transition occurs before the completion of a typical initiation phase (e.g., a full 7 days for apixaban or 21 days of rivaroxaban), then the higher total daily dose of these oral medications is given to complete that initiation phase duration. Some clinicians will transition to the treatment phase dosing of oral anticoagulants if at least 5 days of parenteral heparin have been given, even if this strategy was not tested in the phase 3 randomized trials or included in the package label dosing recommendations.

For patients in the outpatient setting, direct oral anticoagulants (DOACs) with apixaban and rivaroxaban are effective oral-only options for patients who do not want parenteral lead in therapy (Figure 1). No single DOAC is recommended over another by most major society guidelines [5,7,8]. Apixaban and rivaroxaban are appropriate for use in patients with obesity with a BMI > 40 kg/m^2^ or a weight >120 kg. Cost should be considered for DOAC therapy, which can be a barrier for patients. However, there are assistance programs available from drug manufacturers that can substantially reduce the out-of-pocket cost for many patients. Discussion with pharmacists and/or social workers is often helpful to connect patients with appropriate resources for DOAC coverage.

### 3.2. Treatment Phase

The treatment phase can last between 3 and 6 months, depending on the thrombus burden, symptoms, and patient clinical scenario. The American Society of Hematology recommends that this treatment phase last only 3–6 months rather than a more extended duration of 12 months [7]. DOACs are now the mainstay treatment in this phase. However, vitamin K antagonists (VKAs) are an acceptable alternative for most patients and may be preferred in selected patient groups (see below).

For patients using apixaban or rivaroxaban as an oral-only strategy during the initiation phase, these DOACs are typically continued into the treatment phase, but with a dose reduction (Figure 1). Dabigatran and edoxaban, on the other hand, are initiated in the treatment phase after a 5–10-day run-in period (initiation phase) with a parenteral anticoagulant. VKA with warfarin continues to be a well-studied anticoagulation option, though it can be difficult, requiring frequent lab work and a higher risk of bleeding. Warfarin needs to be monitored through the international normalized ratio (INR) with a goal of INR 2–3. There can be higher variability amongst patients for warfarin dosing given patient-specific factors such as diet, genetics, or other medications. Pharmacy costs of warfarin can be lower than DOACs for many patients, but the cost of INR laboratory testing or home testing must also be factored into the overall cost estimates. Most patients are started on warfarin 5 mg daily, with frequent INR testing at least weekly to help determine the warfarin dosing regimen. The mechanism of action plus special considerations for oral anticoagulation are outlined in Table 2. Overall, the treatment of choice for anticoagulation should be patient-specific, with shared decision-making between the patient and provider.

### 3.3. Extended Phase

Extended phase, or anticoagulation beyond the treatment phase of 3–6 months, is considered for certain patient populations depending on the patients’ risk of recurrent VTE versus the risk of bleeding with continued treatment. Patient preference as well as risk scores (e.g., HERDOO2 Rule, Vienna Prediction Model, or DASH Prediction Score) [9,10,11] can assist with the decision-making process for extended-phase anticoagulation. In patients with a low risk of VTE recurrence who had a transiently provoked VTE (Table 1), anticoagulation beyond 3–6 months of the treatment phase is usually not necessary. Generally, for patients with unprovoked VTE, extended-phase treatment should be considered. Both DOACs and warfarin are viable options for extended-phase anticoagulation. For patients continuing on warfarin for VTE prevention, an INR goal of 2–3 is recommended. Dabigatran, apixaban, and rivaroxaban are all potential options for continued anticoagulation for secondary VTE prevention. These three DOACs have been compared to placebo in studies demonstrating superiority in preventing VTE recurrence without significant rates of major bleeding [12,13,14,15].

However, only apixaban and rivaroxaban have demonstrated both efficacy and safety in lower doses than their initial treatment phase doses for recurrent VTE prevention. In AMPLIFY-EXTEND, apixaban 5 mg BID was compared to apixaban 2.5 mg BID and placebo, demonstrating similar rates of recurrent VTE in both apixaban groups and superiority to the placebo group [14]. Patients who had a symptomatic DVT or PE and received treatment for 6–12 months without a recurrent VTE episode were included in the study. The EINSTEIN-CHOICE trial studied rivaroxaban 20 mg daily with rivaroxaban 10 mg daily and aspirin 100 mg daily. Both rivaroxaban groups had similar rates of recurrent VTE and a reduced rate of VTE compared to the aspirin group, while having no significant difference in the rate of major bleeding [15]. Patients who had an objectively confirmed, symptomatic proximal DVT or PE, anticoagulation for 6–12 months, and no interruption in anticoagulation 7 days prior to enrollment were included in the study. Given these studies, lower-dose DOACs compared to standard therapy should be considered for continued anticoagulation in the prevention of recurrent VTE. Once again, this decision is patient-specific, weighing the risk vs. benefit of full vs. reduced DOAC dosing.

The guidelines recommend that patients with recurrent VTE and/or patients with a history of strong thrombophilia be offered extended-phase anticoagulation therapy [5,7]. This recommendation is based on the higher risk of VTE recurrence, which outweighs the risk of bleeding with extended anticoagulation therapy.

## 4. Special Considerations

Special circumstances need to be considered when deciding on management for VTE. Briefly, below, we will review VTE management in cancer-associated thrombosis (CAT), antiphospholipid syndrome (APS), coronary artery disease (CAD), and COVID-19.

### 4.1. Cancer-Associated Thrombosis Treatment

Cancer is among the most common risk factors for VTE, with approximately 20% of all VTE cases occurring in patients with cancer. In these patients, more than 50% of VTE cases occur within 3 months of the cancer diagnosis. Both the American College of Chest Physicians (ACCP) and ASH have guideline recommendations specifically on the management of CAT. ACCP guidelines recommend DOAC over other anticoagulation for acute VTE in the cancer setting [5], while for ASH, both DOACs (apixaban or rivaroxaban) or low-molecular-weight heparin (LMWH) are recommended. The SELECT-D study examined rivaroxaban vs. dalteparin monotherapy and found that the DOAC group at 6 months had significantly fewer recurrent VTE episodes but higher rates of bleeding [16]. The ADAM VTE and Caravaggio studies both looked at apixaban vs. dalteparin, noting a lower risk of recurrent VTE, while the Caravaggio study found no difference in the major bleeding risk [17,18]. For short-term treatment (3–6 months), DOAC is recommended over LMWH. In patients with active cancer and VTE, long-term anticoagulation is recommended for secondary prophylaxis, which can be achieved through DOAC or LMWH. In patients with cancer and recurrent VTE on anticoagulation, an inferior vena cava filter is not recommended [19]. These recommendations should be considered when treating VTE in patients with cancer-associated thrombosis. It is worth noting that special considerations should be made for gastrointestinal or genitourinary malignancies, as in these select populations, DOACs have demonstrated higher rates of bleeding [20]. Additionally, there should be close communication with the patient’s oncologist given drug–drug interactions with DOACs and cancer therapies. DOAC uptake is dependent on the P-glycoprotein system, while metabolism is dependent on the cytochrome P450 system. DOACs should be avoided when co-administrated with cancer therapies that are strong P-glycoprotein or CYP3A4 inducers or inhibitors [21].

### 4.2. Thrombophilia and Antiphospholipid Syndrome Treatment

Warfarin and other VKAs have been the mainstream treatment for thrombotic antiphospholipid syndrome (APS). However, given the increased use of DOACs for other conditions, the use of DOACs for APS remains controversial. While DOACs are far more convenient for patients and are associated with lower rates of bleeding than VKA, it is unclear if they are as effective as VKA in patients with APS. Khairani et al. conducted a systematic review and meta-analysis of RCTs comparing DOACs vs. VKA for the treatment of VTE in patients with APS. Four open-labeled RCTs were included, as summarized in Table 3 [22,23,24,25,26]. The study found that DOACs, compared to VKA, have an increased risk of arterial thrombosis but a similar risk of subsequent VTE or major bleeding. Overall, the findings did not support the routine use of DOACs for patients with thrombotic APS [22]. Additionally, all major societal guidelines recommend the use of VKA over DOACs for APS [5,7,8]. However, there may still be select cases where DOAC therapy is appropriate for a patient with APS, especially if that strongly aligns with the patient’s values/preferences and they are well informed of the current outcome data. In particular, patients with only one or two positive antibodies, patients who have previously tolerated VKA therapy, and/or patients who express a strong preference for VKA therapy over DOAC may be appropriate for VKA therapy. However, it is important for clinicians to engage in a shared decision–discussion with the patient before selecting DOAC over VKA therapy.

Patients with other thrombophilias can be safely treated with DOAC therapy. In a meta-analysis of randomized trials, patients with thrombophilia had similar rates of recurrent VTE and bleeding when treated with DOAC as a VKA therapy [27]. However, care must be taken when ordering and interpreting thrombophilia laboratory tests while being treated with DOAC therapy, as many anticoagulants can interfere with thrombophilia testing processes [28].

### 4.3. Concurrent Coronary Artery Disease and Venous Thromboembolism

The optimal antithrombotic regimen can be difficult to determine for patients with both CAD and VTE. Historically, patients have been treated with triple therapy, including two antiplatelet agents (low-dose aspirin and P2Y12 inhibitors) and anticoagulation. However, this triple therapy combination increases the risk of bleeding up to 3-fold compared to oral anticoagulation alone [29]. Studies examining the risk of bleeding on oral anticoagulation have demonstrated lower rates of bleeding in patients with VTE as compared to those with AF, likely due to their younger age and fewer comorbidities. However, several key factors are critical to consider when a patient on anticoagulation for VTE undergoes percutaneous coronary intervention (PCI). These include the planned duration of anticoagulation, the urgency of PCI, and how best to combine anticoagulation with anti-platelet therapy to decrease bleeding risk.

**Figure 2:**
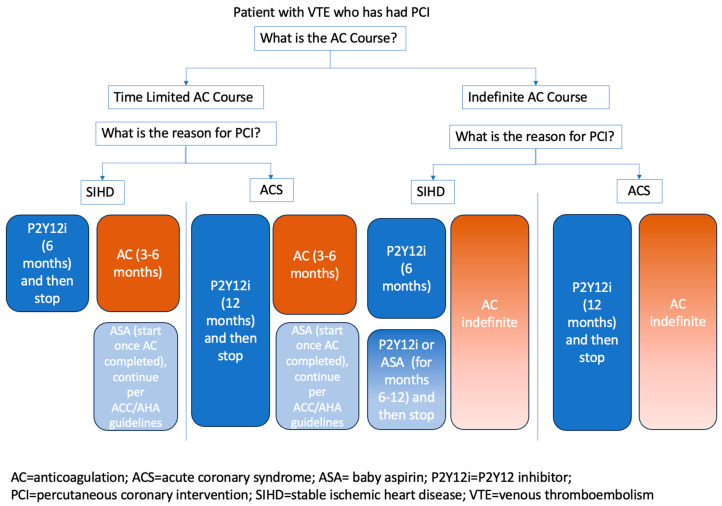
VTE and CAD antithrombotic therapy.

The American College of Cardiology (ACC) has developed clinical pathways to assist with anticoagulation and antiplatelet therapy. The first key distinction to make is the duration of anticoagulation and if it will be indefinite therapy, as discussed prior. Next, the reason for PCI (stable ischemic heart disease vs. acute coronary syndrome (ACS)) divides the pathways in length and choice of antiplatelet therapy. (Figure 2). Finally, all patients should be started on proton pump inhibitors or H2 blockers to decrease the risk of bleeding when they are using multiple antithrombotic agents concurrently [30]. The use of DOAC is preferred over warfarin while on antiplatelet therapy, given the lower risk of major, intracranial, or fatal bleeding with DOAC therapy. However, special considerations (e.g., the use of warfarin for APS) must be taken into account for individual patients [30].

### 4.4. COVID-19 Infection

The COVID-19 infection creates a pro-inflammatory state that often increases a patient’s risk for VTE, especially when the infection is severe enough to require hospitalization. For patients who develop VTE concurrently with a COVID-19 acute infection, standard anticoagulation therapy as outlined above is recommended. These patients are typically considered to have experienced a transient, reversibly provoked VTE, so a shorter course of 3–6 months of anticoagulation is most common [31].

The pro-inflammatory and thrombotic nature of COVID-19 has led to evolving recommendations regarding the use of anticoagulation for VTE thromboprophylaxis. The recommendations require a balance between thrombotic risk and bleeding risk, as well as the patient’s overall risk of survival. In general, patients with COVID-19 can be categorized into one of three groups: ambulatory, hospitalized non-critically ill, and hospitalized critically ill. Based on the results of several randomized trials in patients who require oxygen but are not critically ill (i.e., not in intensive care), a therapeutic dose of heparin (preferentially LMWH) is recommended for patients with D-dimer above the upper limit of normal and without increased bleeding risk [31,32,33,34,35]. These patients should continue therapeutic-intensity thromboprophylaxis for 14 days or until discharge/escalation of care to an intensive care unit. All other hospitalized patients should receive standard VTE thromboprophylaxis with prophylactic doses of heparin. The use of DOACs for inpatient thromboprophylaxis is generally not recommended [35,36]. However, consideration can be made for the use of extended post-hospital thromboprophylaxis with low-dose rivaroxaban in select patients at high thromboembolic risk but low bleeding risk [37]. Finally, antiplatelet therapy to prevent COVID progression or death is not recommended based on the negative results of the ACTIV-4a and RECOVERY trials [32,33,34,35,36,37,38].

## 5. Future Anticoagulation Options

Anticoagulation management for VTE looks different today than twenty years ago. DOACs revolutionized VTE management with their increased ease of administration and lower risk of bleeding, but they are more limited in terms of clinical applications. There continue to be ongoing clinical trials examining new anticoagulation medications. Table 4 summarizes the current ongoing trials for VTE and Factor XI/XIa inhibitors [39,40,41]. These agents might allow for further reductions in the bleeding risk by uncoupling thrombosis and hemostasis. Furthermore, they may provide further advantages over DOACs by eliminating concerns about renal clearance and longer half-lives to address issues of medication compliance. However, their efficacy in preventing VTE or VTE recurrence remains to be proven in rigorous phase 3 randomized trials.

## 6. Final Thoughts

VTE is a highly prevalent condition associated with significant morbidity and mortality. While anticoagulation is the mainstream therapy for VTE, the decision on when, how, and for how long to administer anticoagulants is increasingly complex. By considering each individual patient’s underlying thromboembolic and bleeding risk, clinicians can then apply evidence from both randomized and observational data to personalize anticoagulation therapy. Anticoagulation management will continue to evolve with new agents and new evidence that aim to maximize effectiveness and minimize risk.

## Figures and Tables

**Table 1:** Examples of VTE provoking risk factors.

Major Transient Risk Factors	Minor Transient Risk Factors	Persistent Risk Factors
-Cesarean section. -Confined to hospital bed for 3 days. -Surgery with general anesthesia for >30 min.	-Confined to bed out of hospital for 3 days. -Hospitalization < 3 days. -Leg injury. -Pregnancy. -Estrogen therapy. -Acute infectious illness (e.g., COVID-19) without hospitalization.	-Active cancer. -Inflammatory bowel disease. -Obesity. -Chronic inflammatory condition. -Advanced age. -Previous venous thromboembolism. -Genetic/acquired thrombophilia (APS, protein C&S deficiency, etc.).

**Table 2:** Oral anticoagulation for VTE.

Generic Name	Mechanism of Action	Dose and Regimen	Consideration of Renal Function	Consideration of Drug Interactions	Other Considerations
*Apixaban*	Factor Xa Inhibitor	10 mg BID × 7 days, followed by 5 mg BID	Not studied in patients with SCr ≥ 2.5 mg/dL or CrCl <25 mL/min	Reducing dose by 50% in patients taking strong dual *inhibitors* of p-glycoprotein and CYP 3A4. Avoiding in patients taking dual *inducers* of CYP 34A and p-glycoprotein.	N/a
*Dabigatran*	Direct Thrombin Inhibitor	150 mg BID after 5–10 days of parenteral anticoagulation lead in	Avoid in CrCl ≤ 30 mL/min	If CrCl ≤ 50 mL/min, patients taking p-glycoprotein *inhibitors* should avoid dabigatran. Patients taking p-glycoprotein *inducers* should avoid dabigatran.	N/a
*Edoxaban*	Factor Xa Inhibitor	60 mg daily after 5–10 days of parenteral anticoagulation lead in	Renally dose to 30 mg daily for CrCl 15–50 mL/min. Avoid in CrCl <15 mL/min	Reduce dose to 30 mg daily for patients taking p-glycoprotein *inhibitors*. Avoid using with p-glycoprotein *inducers*.	Reduce dose to 30 mg daily for body weight ≤ 60 kg.
*Rivaroxaban*	Factor Xa Inhibitor	15 mg twice a day for 21 days, then 20 mg daily	Avoid in CrCl ≤ 15 mL/min	In patients taking moderate dual *inhibitors* of CYP 3A4 and p-glycoprotein with CrCl ≤ 80 mL/min, use cautiously. Avoid use in patients taking strong dual *inhibitors* or *inducers* of CYP 3A4 and p-glycoprotein.	Administer with food.
*Warfarin*	Vitamin K Antagonist	Adjusted to target INR 2–3 Require parenteral anticoagulation overlap at initiation	None	Consider reducing starting dose to 2.5 mg for patients with drug–drug interactions expected to increase exposure to warfarin.	Consider reducing starting dose to 2.5 mg for patients with multiple comorbidities, advanced age, and advanced end-organ dysfunction.

BID = twice daily; CrCl = creatinine clearance as calculated by the Cockcroft–Gault equation with actual body weight; INR = international normalized ratio; N/a = not applicable; SCr = serum creatinine.

**Table 3:** Randomized trials of oral anticoagulation for antiphospholipid syndrome patients with venous thromboembolism.

Clinical Trial (Ref. #)	Included Patients	N	Trial Design	Length of Follow-Up	Treatment Groups	Primary Efficacy Outcomes	Efficacy Outcomes	Major Bleeding Outcomes
RAPS [23]	Patients with APS who were taking warfarin for previous VTE	116	Open-label RCT	210 days	Continue warfarin vs. rivaroxaban 20 mg daily	Percentage change in endogenous thrombin potential at day 42, with non-inferiority set at less than 20% difference from warfarin	ETP (nmol/L per min): Rivaroxaban 1086 vs. warfarin 548 Treatment effect (ratio): 2.0 (1.7–2.4)	Rivaroxaban: 0 Warfarin: 0
TRAPS [24]	Patients with APS (triple positivity) with history of thrombus	120	Open-label RCT	569 days (mean)	Rivaroxaban 20 mg or 15 mg daily (dependent on creatine clearance) vs. warfarin	Cumulative incidence of thromboembolic events, major bleeding, and vascular death	Rivaroxaban: 19% Warfarin: 3% HR: 6.7 (1.5–30.5)	Rivaroxaban: 7% Warfarin: 3% HR: 2.5 (0.5–13.6)
Ordi-Ros et al. [25]	Patients with APS (positive result on aPL testing on 2 occasions at least 3 months apart) with history of thrombus	190	Open-label RCT	36 months	Rivaroxaban 20 mg or 15 mg daily (dependent on creatine clearance) vs. warfarin	Proportion of patients with new thrombotic event	Rivaroxaban: 11.6% Warfarin: 6.3% HR: 1.94 (0.72–5.24)	Rivaroxaban: 6.3% Warfarin: 7.4% HR: 0.88 (0.3–2.63)
ASTRO-APS [26]	Patients with thrombotic antiphospholipid syndrome on anticoagulation for secondary prevention	48	Open-label RCT	12 months	Apixaban 2.5 mg BID then increased to 5 mg BID (after 25 patient was randomized) vs. warfarin	Thrombosis and vascular death	Apixaban: 6 thrombotic events Warfarin: no thrombotic events	Apixaban: 0 Warfarin: 1 event

APS = antiphospholipid syndrome; BID = twice daily; ETP = endogenous thrombin potential; HR = hazard ratio; RCT = randomized control trial; VTE = venous thromboembolism.

**Table 4:** Factor XI ongoing clinical trials for VTE.

Clinical Trial Reference (Status)	Drug	Mechanism of Action	N	Clinical Trial Summary	Results
ASTER NCT05171049 (Ongoing) [39]	Abelacimab	Binds and inhibits Factor XI and Factor XIa	1655	Phase III trial comparing the effect of abelacimab relative to apixaban on VTE recurrence and bleeding in patients with CAT	No results currently
MAGNOLIA NCT05171075 (Ongoing) [40]	Abelacimab	Binds and inhibits Factor XI and Factor XIa	1020	Phase III trial comparing the effect of abelacimab vs. dalteparin on VTE recurrence and bleeding in patients with gastrointestinal or genitourinary CAT	No results currently
NCT04465760 (Recruiting) [41]	Xisomab	Binds Factor XI and blocks activation by Factor XIIa	50	Phase II trial examining the efficacy of xisomab as measured by incidence of catheter associated thrombosis in individuals with a central venous catheter	No results currently

CAT = cancer-associated thrombosis; VTE = venous thromboembolism.

## Data Availability

Not applicable.
